# Mineralocorticoid Receptor-Dependent Impairment of Baroreflex Contributes to Hypertension in a Mouse Model of Primary Aldosteronism

**DOI:** 10.3389/fphys.2019.01434

**Published:** 2019-11-22

**Authors:** Luo Shi, Fang Yuan, Xuefang Wang, Ri Wang, Kun Liu, Yanming Tian, Zan Guo, Xiangjian Zhang, Sheng Wang

**Affiliations:** ^1^Department of Physiology, Hebei Medical University, Shijiazhuang, China; ^2^Department of Physiology, Hebei North University, Zhangjiakou, China; ^3^Department of Laboratory Medicine, Hebei University of Chinese Medicine, Shijiazhuang, China; ^4^Hebei Key Laboratory of Vascular Homeostasis and Hebei Collaborative Innovation Center for Cardio-Cerebrovascular Disease, Second Hospital of Hebei Medical University, Shijiazhuang, China

**Keywords:** aldosterone, baroreflex, hypertension, mineralocorticoid receptor, aldosteronism

## Abstract

Primary aldosteronism (PA) is the most common cause of secondary hypertension. The paucity of good animal models hinders our understanding of the pathophysiology of PA and the hypertensive mechanism of PA remains incompletely known. It was recently reported that genetic deletion of TWIK-related acid-sensitive potassium-1 and potassium-3 channels from mice (TASK^−/−^) generates aldosterone excess and mild hypertension. We addressed the hypertensive mechanism by assessing autonomic regulation of cardiovascular activity in this TASK^−/−^ mouse line that exhibits the hallmarks of PA. Here, we demonstrate that TASK^−/−^ mice were hypertensive with 24-h ambulatory arterial pressure. Either systemic or central blockade of the mineralocorticoid receptor (MR) markedly reduced elevated arterial pressure to normal level in TASK^−/−^ mice. The response of heart rate to the muscarinic cholinergic receptor blocker atropine was similar between TASK^−/−^ and wild-type mice. However, the responses of heart rate to the β-adrenergic receptor blocker propranolol and of arterial pressure to the ganglion blocker hexamethonium were enhanced in TASK^−/−^ mice relative to the counterparts. Moreover, the bradycardiac rather than tachycardiac gain of the arterial baroreflex was significantly attenuated and blockade of MRs to a large degree rescued the dysautonomia and baroreflex gain in TASK^−/−^ mice. Overall, the present study suggests that the MR-dependent dysautonomia and reduced baroreflex gain contribute to the development of hyperaldosteronism-related hypertension.

## Introduction

Primary aldosteronism (PA) is one of the most common causes of secondary hypertension, accounting for ~10% of hypertensive patients and ~20% of resistant hypertension (Douma et al., 2008). It is characterized by high plasma aldosterone (Aldo), reduced plasma renin activity, varying degrees of hypokalemia, metabolic alkalosis and mild hypertension. Compared with essential hypertension, patients with PA manifest higher cardiovascular morbidity and mortality (Mulatero et al., 2013). However, the hypertensive mechanism of PA remains incompletely understood. The moderate increase in blood volume caused by Aldo-stimulated sodium reabsorption and water retention seems inadequate to explain the pathophysiological mechanism of elevated arterial pressure (Weiner, 2013).

Aldo, produced by the adrenal zona glomerulosa (ZG) cells, plays a fundamental role in the maintenance of salt and water homeostasis. Despite the evidence of contribution of G protein-coupled estrogen receptor activation to the non-genomic effects of Aldo (Brailoiu et al., 2013), the majority of Aldo’s effects are exerted through the activation of the mineralocorticoid receptor (MR) (Hermidorff et al., 2017). Accumulated evidence has implicated the importance of Aldo/MR signaling in the pathogenesis of different hypertensive models, including renovascular hypertension (Lincevicius et al., 2015), stroke-prone spontaneous hypertension (Nakagaki et al., 2012) and high salt-loaded hypertension (Masuda et al., 2010). Chronic systemic administration of Aldo/deoxycorticosterone (DOC) elevated arterial pressure in several animal models (Garwitz and Jones, 1982; Chen et al., 1989; Xue et al., 2012). Intracerebroventricular (ICV) infusions of Aldo/DOC at doses that were ineffective when infused systemically were found to raise arterial pressure, and this central pressor effect was blocked by ICV infusion of MR antagonists (Gomez-Sanchez, 1986; Gomez-Sanchez and Gomez-Sanchez, 2012). Moreover, both acute and chronic administration of Aldo impaired baroreflex in animals (Wang, 1994) and healthy humans (Heindl et al., 2006; Monahan et al., 2007). Nevertheless, these mechanisms are insufficient to elucidate the onset and development of hyperaldosteronism-related hypertension.

Recently, global deletion of TWIK-related acid-sensitive potassium-1 (TASK-1) and TASK-3 channels from mice led to membrane depolarization of adrenal ZG cells and elicited autonomous Aldo overproduction and attendant hypertension. In addition, Aldo excess was independent of enhanced activity of the renin-angiotensin system and failed to decrease in response to dietary salt loading (Davies et al., 2008). Thus, the TASK channel knockout (TASK^−/−^) mouse line represents an animal model of PA that exhibits the particular hallmarks of idiopathic primary aldosteronism. More recently, selective deletion of TASK channels from mouse ZG cells also produced mild hyperaldosteronism with attendant hypertension (Guagliardo et al., 2019). These findings provide suitable animal models of PA to unveil hypertensive mechanism. The arterial baroreflex is a crucial homeostatic mechanism of the regulation of cardiovascular activity (Landgren, 1952; Heymans, 1960). Chronic impairment of this reflex results in a sustained increase in sympathetic activity with serious consequences in cardiovascular diseases, such as hypertension, heart failure, and stroke (Mortara et al., 1997; La Rovere et al., 2001; Robinson et al., 2003). Hence, whether the dysfunctional arterial baroreflex promotes hyperaldosteronism-associated hypertension is of particular interest and importance, which requires further investigation that could be translated into more assertive therapeutic strategies.

We therefore used the TASK^−/−^ mouse line as a PA model and aimed to address the putative mechanism underlying hyperaldosteronism-associated hypertension. To that end, we sought to test whether autonomic control of blood pressure (BP) and heart rate (HR) differed between wild-type and TASK^−/−^ mice, to test whether the arterial baroreflex function was impaired in TASK^−/−^ mice, and to test whether the activation of MR was required for dysautonomia and dysfunctional baroreflex in TASK^−/−^ mice.

## Materials and Methods

### Experimental Mice

Animals were housed individually and allowed to move freely in standard plastic cages under 12-h light/dark cycles (7:00–19:00 light, 19:00–7:00 dark) at standard laboratory conditions (temperature 22 ± 1°C and humidity 50 ± 10%). Food and water were provided *ad libitum*. TASK^−/−^ mice were kindly presented by Dr. Douglas Bayliss from University of Virginia and have been validated previously (Mulkey et al., 2007). All aged-matched male TASK^−/−^ and C57BL/6 (TASK^+/+^, for control) mice (12–15 weeks old, 23–30 g) were used in this study. All experiments were conducted in accordance with Guide for the Care and Use of Laboratory Animals, and were approved by Animal Care and Ethical Committee of Hebei Medical University.

### Telemetric Recordings of Blood Pressure and Heart Rate in Conscious Mice

BP and HR were measured in conscious, freely moving TASK^−/−^ mice and the counterparts using telemetry system (Ponemah v6.00, Data Sciences International, USA). In this system, the transmitter (HD-X11) was allowed to transfer the signal to a remote receiver and a data-exchange matrix (PhysioTel Matrix 2.0) connected to a computer. Mice were inhalationally anesthetized with isoflurane (2~3%, RWD Life Science) after initial exposure to a chamber filled with isoflurane for 1~2 min. Depth of anesthesia was assessed by absence of corneal and hindpaw withdrawal reflexes. All surgical procedures were conducted under strict aseptic conditions. After anesthesia, the pressure catheter was inserted into the right common carotid artery and the transmitter body was placed subcutaneously in the abdominal area. After wound closure, the mice received injections of antibiotic ampicillin (125 mg/kg, i.p.) and the analgesic ketorolac (4 mg/kg, i.p.). At least 5 days after recovery, systolic blood pressure (SBP), diastolic blood pressure (DBP), and HR were measured every 1 h for 24 h at a sample rate of 1 kHz.

### Blockade of Mineralocorticoid Receptors

For systemic blockade of MRs, the competitive MR antagonist spironolactone (SPIR, Hangzhou Minsheng Pharmaceuticals, Hangzhou, China) was dissolved in water and administered *via* oral gavage at 100 mg/kg once daily for seven consecutive days as described previously (Ahokas et al., 2003). For central blockade of MRs, an ICV injection was carried out in two genotypes. In brief, mice were anesthetized with pentobarbital (50 mg/kg, i.p.) and positioned in a stereotaxic instrument (RWD Life Science, China) with the skull leveled between bregma and lambda. A stainless-steel cannula was inserted into the lateral ventricle (0.5 mm posterior to bregma, 1.0 mm lateral to the midline, 2.5 mm ventral to the skull surface) and fixed to the skull surface using screws and dental acrylic. A dummy cannula was inserted into the guide cannula to prevent infection, exposure, or occlusion. Mice were allowed to recover for at least 1 week. On the day for infusion, the dummy cannula was replaced with an internal cannula in inhalationally anesthetized mice with isoflurane (2~3%). The internal cannula was connected by polyethylene tubing to a syringe filled with SPIR or vehicle. Injections of SPIR (5 μl, 10 μg/kg per day, dissolved in 0.9% saline with 2% ethanol) and equal volume of vehicle were completed in 5 min *via* ICV once daily for 7 days. The dose of SPIR was calculated according to previous studies (Lu et al., 2018). At the end of experiments, an Evans Blue solution was injected in each mouse to ensure the right position of the ICV cannula.

### Evaluation of Sympathetic and Parasympathetic Tone

To assess autonomic function in TASK^−/−^ mice, the responses of HR to the β-adrenergic receptor blocker propranolol (1 mg/kg, i.p., Tocris, USA) and to the cholinergic receptor blocker atropine (1 mg/kg, i.p., Tocris, USA) were measured *via* telemetric system on separate days in conscious awake mice during resting periods. This reduced the effect of locomotor activity on autonomic drive and allowed a more accurate estimate of resting cardiac sympathetic and parasympathetic tones. To examine the neurogenic contribution to elevated arterial pressure in TASK^−/−^ mice, hexamethonium bromide (5 mg/kg, i.p., Tocris, USA), a ganglion blocker, was given in conscious mice. BP was monitored and comparisons were made between two genotypes. The dose of atropine, propranolol, and hexamethonium bromide has been validated previously (Lu et al., 2009).

### Analysis of Baroreflex Function

The baroreflex function was assessed pharmacologically in anesthetized mice (urethane at 800 mg/kg and α-chloralose at 40 mg/kg). Shortly, a cannula was inserted into the jugular vein and a tracheostomy was performed. A cannula filled with heparin-saline was inserted in the carotid artery. BP and HR were recorded consecutively at 1 kHz using a pressure transducer connected to Powerlab System (AD Instruments, Canada). After postsurgical equilibration, phenylephrine (PE, 10, 20, and 40 μg/kg) and sodium nitroprusside (SNP, 30, 60, and 120 μg/kg) were intravenously injected in random order, to evoke acute increases and decreases in BP, respectively. For each injection, the change in HR at the time of peak SBP was chosen and ΔHR/ΔSBP was used as an index of baroreflex gain.

### Measurement of Plasma Aldosterone

The mouse tail arteriovenous mixed blood was collected using an anticoagulant capillary tube at 6:00, 12:00, 18:00, and 24:00, and then centrifuged immediately (1,000×*g* for 15 min) at 4°C with a hematocrit centrifuge (CENCE, China). The plasma samples were stored at −20°C until experimental day. The Aldosterone Parameter Assay Kit (KGE016, R&D Systems) was used to measure plasma aldosterone concentration according to the manual.

### Statistics

Data were expressed as mean ± standard error. Statistical analysis was made using Prism (GraphPad Prism, USA). To compare the differences, we used unpaired *t* test, one- or two-way ANOVA with appropriate *post hoc* test and Kruskal-Wallis test followed by Dunn’s multiple test as indicated in the context. Differences with *p* < 0.05 were considered statistically significant, and the number of comparisons was indicated in the figure legends.

## Results

### Cardiovascular Phenotype in TASK^−/−^ Mice

**Figure 1:**
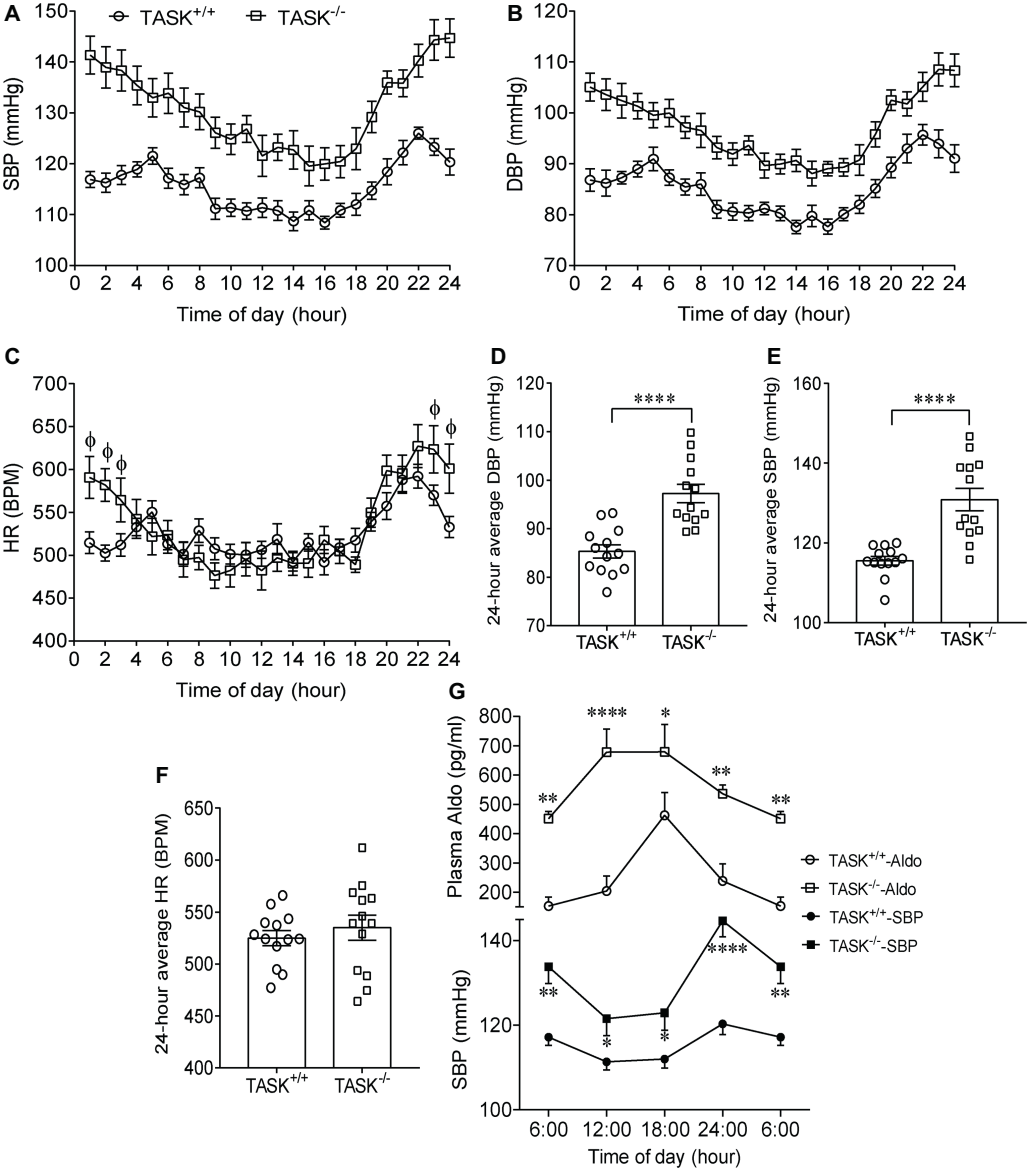
Hypertension in TASK^−/−^ mice. Continuous telemetric recordings of SBP, DBP, and HR over 24-h period were performed in TASK^+/+^ and TASK^−/−^ mice (*n* = 13 for each genotype). Measurements were made hourly over 24 h. **(A–C)** Dynamic SBP, DBP, and HR over 24 h. SBP and DBP were significantly higher at each time point over 24 h in TASK^−/−^ mice compared to TASK^+/+^ mice (*p* < 0.05, two-way ANOVA with Fisher’s LSD *post hoc* test). HR measured over 24 h in TASK^−/−^ mice was not different from control mice, except for higher HR at specific time points (*p* < 0.05 as indicated by Φ, two-way ANOVA with Fisher’s LSD *post hoc* test). **(D–F)** 24-h average SBP, DBP, and HR. ^****^p < 0.0001 as indicated, unpaired *t* test. **(G)** Circadian changes in plasma Aldo and arterial pressure. Top panel: plasma level of Aldo was measured at 6:00, 12:00, 18:00, and 24:00. *n* = 16 mice for each genotype. Bottom panel: telemetric recordings of SBP. *n* = 13 mice for each genotype. ^*^p < 0.05, ^**^p < 0.01, ^****^p < 0.0001 by two-way ANOVA with Fisher’s LSD *post hoc* test, TASK^−/−^ versus TASK^+/+^ mice. SBP, systolic blood pressure; DBP, diastolic blood pressure; HR, heart rate; BPM, beat per minute.

**Figure 2:**
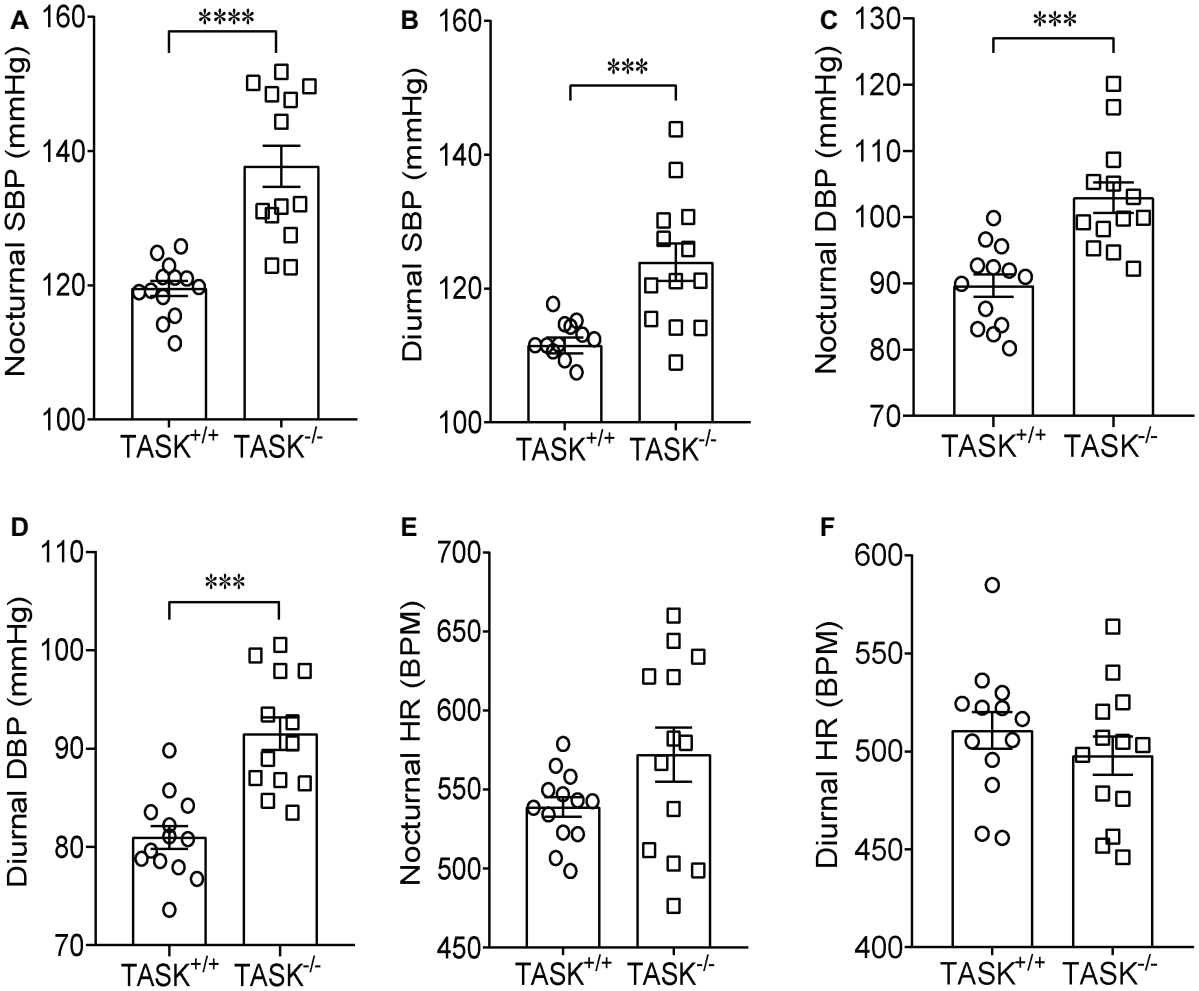
Higher nocturnal and diurnal arterial pressure in TASK^−/−^ mice. Telemetric recordings of arterial pressure and HR were made over 24 h in TASK^+/+^ and TASK^−/−^ mice. Nocturnal BP and HR were calculated based on measurements made from 7 pm to 7 am, so 7 am to 7 pm for diurnal BP and HR. The average nocturnal SBP **(A)** and DBP **(C)**, diurnal SBP **(B)** and DBP **(D)** were higher in TASK^−/−^ mice than control mice. The average nocturnal **(E)** and diurnal **(F)** HR was similar between two genotypes. *n* = 13 mice for each genotype, ^***^p < 0.001, ^****^p < 0.0001 as indicated by unpaired *t* test.

It has been established that TASK^−/−^ mice exhibit the hallmarks of PA (Davies et al., 2008). Using radio telemetry, we recorded BP and HR between TASK^−/−^ and TASK^+/+^ mice in conscious and freely moving state. TASK^−/−^ mice had a sustained increase in arterial pressure and were hypertensive with 24-h ambulatory SBP and DBP (*p* < 0.05 for each time point, Figures 1A,B), 24-h average SBP and DBP (SBP: 116 ± 1 versus 131 ± 3 mmHg; DBP: 85 ± 1 versus 97 ± 2 mmHg, TASK^+/+^ versus TASK^−/−^, *n* = 13 for each group, *p* < 0.0001 for both, Figures 1D,E). The 24-h ambulatory HR in TASK^−/−^ mice was not different from TASK^+/+^ mice, with the exception of higher HR in TASK^−/−^ mice at specific time points (11 PM to 3 AM local time, Figures 1C,F). Although the circadian rhythm of plasma Aldo and SBP was not exactly parallel in both genotypes, plasma Aldo and the corresponding SBP were always higher at given time points in TASK^−/−^ mice relative to TASK^+/+^ mice (Figure 1G). TASK^−/−^ mice had greater nocturnal and diurnal SBP (Figures 2A,B) and DBP (Figures 2C,D) in comparison to the counterparts. However, no significant difference in nocturnal and diurnal HR was observed between two genotypes (Figures 2E,F).

### Antihypertensive Effects of Mineralocorticoid Receptor Blockade in TASK^−/−^ Mice

**Figure 3:**
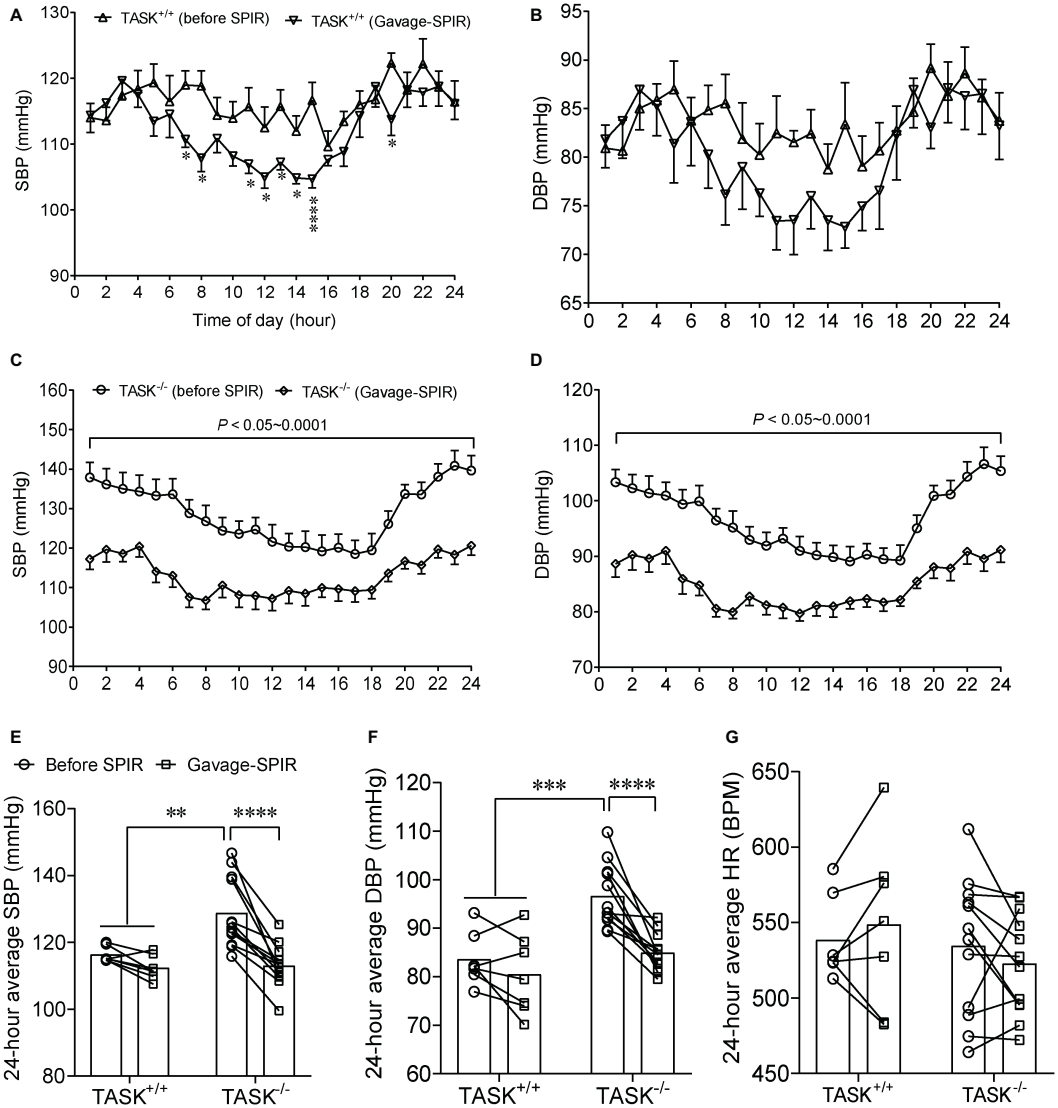
Effect of oral administration of SPIR on arterial pressure. The MR antagonist SPIR was administered *via* oral gavage once daily for 7 days in TASK^+/+^ (*n* = 7) and TASK^−/−^ mice (*n* = 12). **(A,B)** Administration of SPIR produced mild decrease in SBP only at specific time points and no obvious decrease in DBP in TASK^+/+^ mice. ^*^p < 0.05, ^****^p < 0.0001 as indicated by two-way ANOVA with Fisher’s LSD *post hoc* test. **(C,D)** After treatment with SPIR, SBP and DBP were significantly lowered at each time point over 24 h in TASK^−/−^ mice. **(E–G)** Application of SPIR markedly lowered 24-h average SBP and DBP in TASK^−/−^ mice, with insignificant changes in TASK^+/+^ mice. No significant difference was observed in HR between two genotypes after MR blockade. ^**^p < 0.01, ^***^p < 0.001, ^****^p < 0.0001 as indicated by two-way ANOVA with Bonferroni’s multiple comparisons test. MR, mineralocorticoid receptors; SPIR, spironolactone.

**Figure 4:**
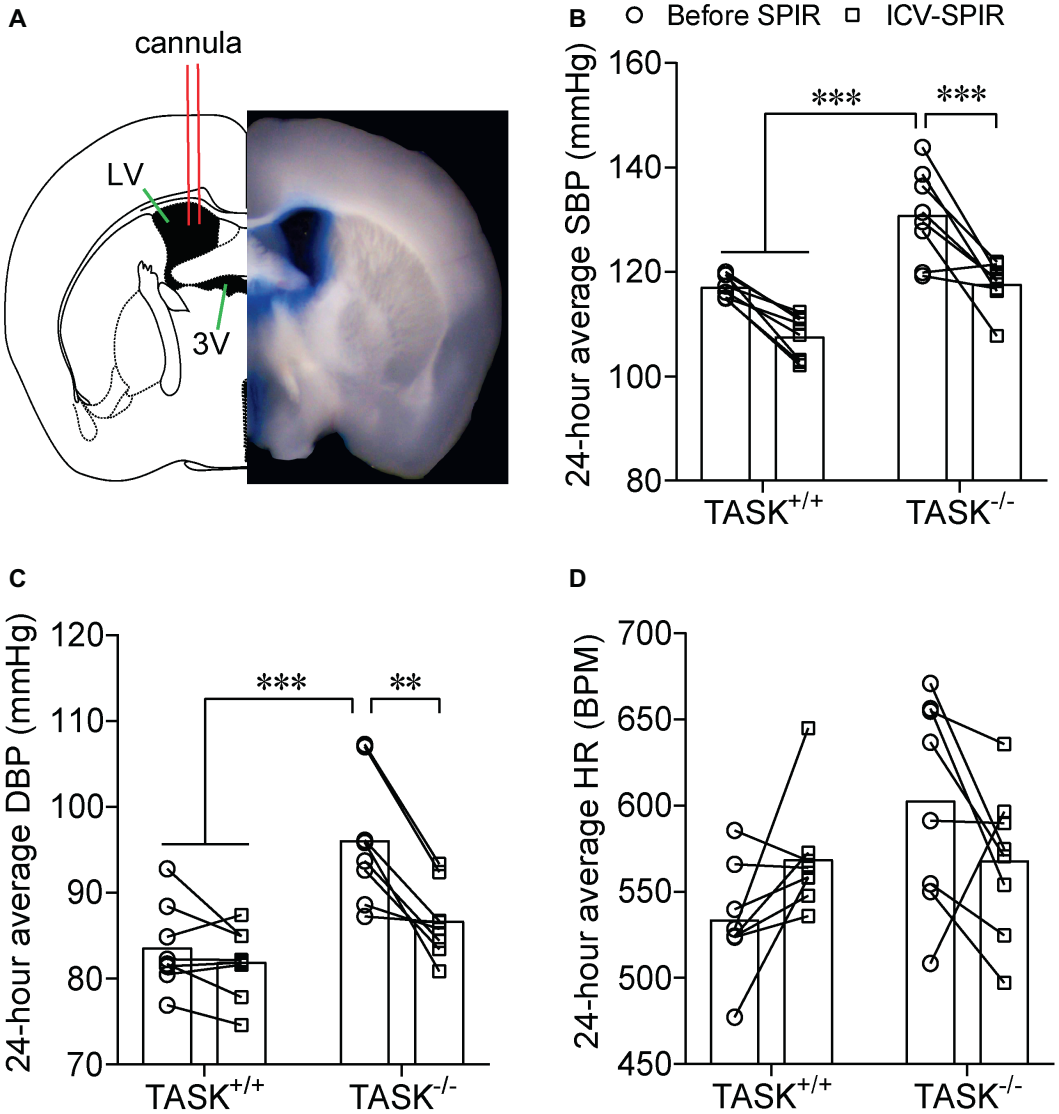
Effect of central MR blockade on arterial pressure. SPIR was centrally given *via* an ICV injection once daily for 7 days in TASK^+/+^ and TASK^−/−^ mice (*n* = 8 for each genotype). **(A)** Shows a schematic diagram of ICV injection (left) and a half slice image (right) of ICV injection of the Evans Blue. Application of SPIR considerably lowered 24-h average SBP **(B)** and DBP **(C)** TASK^−/−^ mice, without significant impact on TASK^+/+^ mice. No obvious difference was found in HR **(D)** in both genotypes. ^**^p < 0.01, ^***^p < 0.001 as indicated by two-way ANOVA with Tukey’s multiple comparisons test. ICV, intracerebroventricular; LV, lateral ventricle; 3V, third ventricle.

Most effects of Aldo are exerted mainly through activation of MRs (Hermidorff et al., 2017). Here, we tested whether the MR was required for hyperaldosteronism-related hypertension in TASK^−/−^ mice. SPIR was administered *via* oral gavage (100 mg/kg/day) once daily for 7 days in both genotypes. In TASK^+/+^ mice, systemic administration of SPIR produced a remarkable decrease in SBP only at specific time points (Figure 3A) and no obvious effect on DBP (Figure 3B). However, in TASK^−/−^ mice, SPIR notably reduced hourly SBP (*p* < 0.05–0.001, Figure 3C) and DBP (*p* < 0.05 ~ 0.001, Figure 3D) over 24 h. Likewise, administration of SPIR significantly decreased 24-h average SBP and DBP in TASK^−/−^ mice (SBP: 129 ± 3 versus 113 ± 2 mmHg; DBP: 97 ± 2 versus 85 ± 1 mmHg, before versus after SPIR, *n* = 12, *p* < 0.0001, Figures 3E,F), with insignificant effects in TASK^+/+^ mice. In addition, no significant change in 24-h average HR was observed between two genotypes (Figure 3G). These data suggest that MR signaling mediates hyperaldosteronism-associated hypertension. To further assess whether central blockade of MRs had an antihypertensive effect, ICV injection of SPIR or equal volume of vehicle (10 μg/kg/day, Figure 4A) was performed once daily for 7 days. As a result, central application of SPIR produced no effect on BP in TASK^+/+^ mice but resulted in significant decreases in 24-h average SBP and DBP in TASK^−/−^ mice (SBP: 131 ± 3 versus 118 ± 2 mmHg; DBP: 96 ± 3 versus 87 ± 2 mmHg, before versus after SPIR, *n* = 8 for both, *p* < 0.001 for SBP, *p* < 0.01 for DBP, Figures 4B,C), with insignificant change in HR in either genotype (Figure 4D), in support of a centrally antihypertensive effect. Note that the injection of vehicle elicited insignificant effects on BP and HR in each genotype (data not shown).

### Mineralocorticoid Receptor-Dependent Dysautonomia in TASK^−/−^ Mice

**Figure 5:**
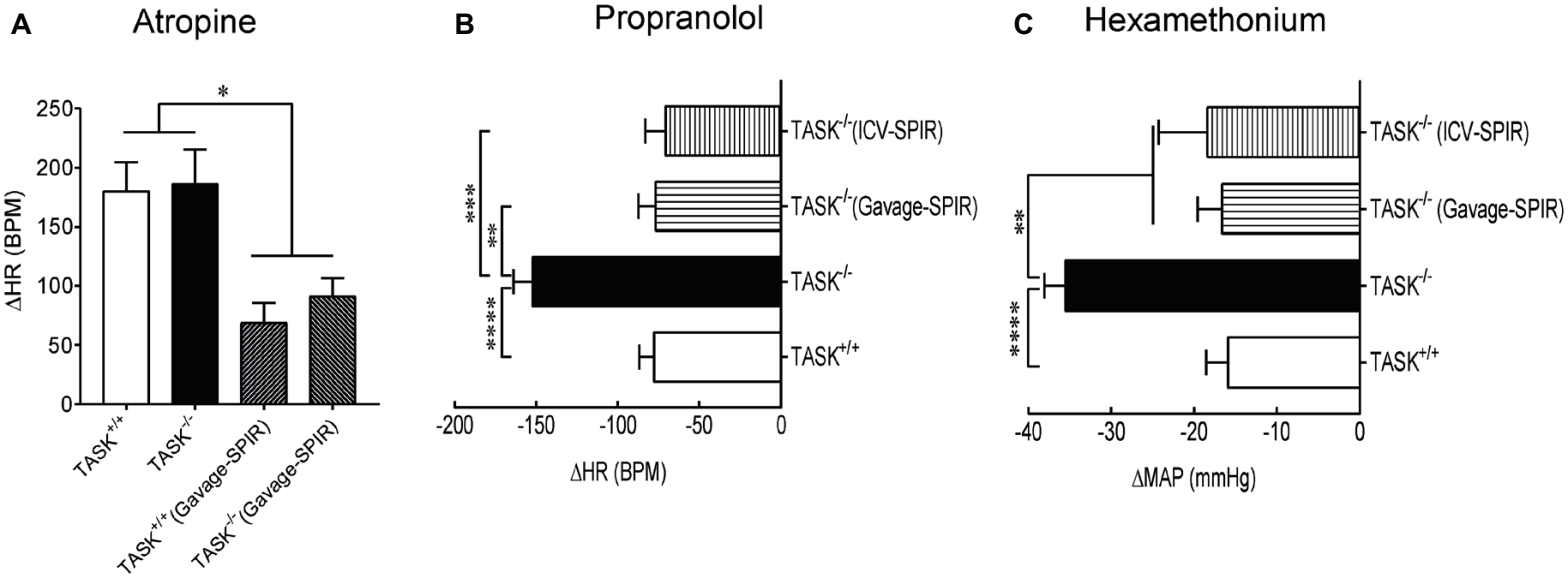
Sympatho-vagal imbalance in TASK^−/−^ mice. **(A)** The tachycardiac response to atropine (ΔHR) was similar between TASK^+/+^ (*n* = 9) and TASK^−/−^ mice (*n* = 7). Systemic blockade of MRs similarly inhibited the tachycardiac response to atropine in two genotypes (*n* = 5 for TASK^+/+^; *n* = 8 for TASK^−/−^). **(B)** The bradycardiac response to propranolol (ΔHR) was greater in TASK^−/−^ mice (*n* = 23) compared to TASK^+/+^ mice (*n* = 19). Either oral gavage (*n* = 7) or ICV injection (*n* = 8) of SPIR significantly diminished the propranolol-stimulated bradycardiac response in TASK^−/−^ mice. **(C)** The decrease in mean arterial pressure (ΔMAP) by a ganglion blockade with hexamethonium was greater in the TASK^−/−^ (*n* = 20) versus TASK^+/+^ (*n* = 16) mice. Such effects were remarkably compromised after either systemic (*n* = 7) or central (*n* = 8) application of SPIR. ^*^p < 0.05, ^**^p < 0.01, ^***^p < 0.001, ^****^p < 0.0001 as indicated by one-way ANOVA with Tukey’s multiple comparisons test.

To address the manifestation of dysautonomia and sympatho-vagal imbalance in conscious TASK^−/−^ mice, pharmacological antagonists were administered in conscious mice to assess reflex control of HR and BP. Intraperitoneal injection of atropine (1 mg/kg) robustly increased HR but no significant change was observed between two genotypes. In addition, there was no difference in atropine-stimulated HR increase between two genotypes after oral administration of SPIR (Figure 5A). These data indicate that the TASK^−/−^ mouse exhibits similarly parasympathetic control of HR to its counterpart. In response to propranolol (1 mg/kg, i.p.), the decrease in HR was almost two-fold greater in TASK^−/−^ mice relative to TASK^+/+^ mice (△HR: −78 ± 9 versus −153 ± 11, TASK^+/+^ versus TASK^−/−^, *n* = 19 for TASK^+/+^, *n* = 23 for TASK^−/−^, *p* < 0.0001, Figure 5B), suggesting an amplified sympathetic control of HR. Both systemic and central blockades of MRs significantly inhibited propranolol-stimulated fall in HR (Figure 5B). While the difference in HR response to propranolol was significant between both genotypes, the corresponding change in BP was variable, small, and not statistically different between the two groups (data not shown).

Next, injection of hexamethonium, a ganglionic blocker, produced a greater decrease in BP in TASK^−/−^ than TASK^+/+^ mice (△MAP: −16 ± 3 versus −36 ± 2, TASK^+/+^ versus TASK^−/−^, *n* = 16 for TASK^+/+^, *n* = 20 for TASK^−/−^, *p* < 0.0001), an effect also abolished by either systemic or central administration of SPIR (Figure 5C). This decrease in BP is indicative largely of the neurogenic and primarily sympathetic contribution to peripheral vascular resistance, further supporting an enhanced sympathetic control of BP in TASK^−/−^ mice. Corresponding reductions in HR were from 637 ± 12 to 602 ± 24 beats/min (*n* = 8) in the TASK^+/+^ mice and from 522 ± 43 to 506 ± 40 beats/min (*n* = 5) in the TASK^−/−^ mice and were not statistically different. Taken together, these results from conscious TASK^−/−^ mice suggest a MR-dependent sympatho-vagal imbalance with augmented sympathetic and unchanged parasympathetic drives.

### Impaired Baroreflex Gain in TASK^−/−^ Mice

**Figure 6:**
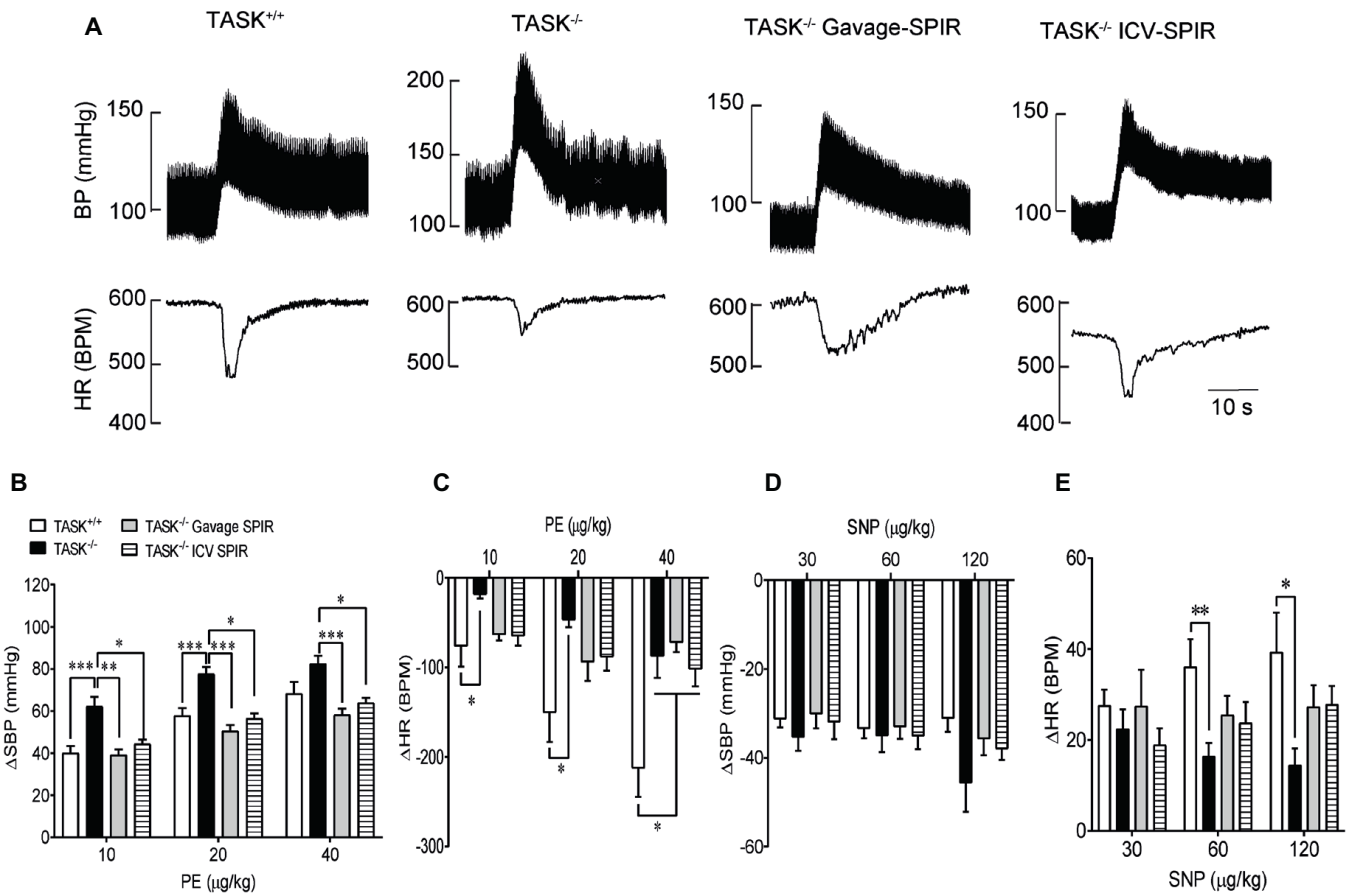
Baroreceptor reflex function. The baroreflex function was measured pharmacologically in anesthetized TASK^+/+^ and TASK^−/−^ mice. **(A)** Typical traces showing responses of BP and HR to intravenous injection of PE. **(B,C)** The mean change in SBP (ΔSBP) and HR (ΔHR) in response to intravenous injection of PE. Statistical analysis reported significant effects of dose and genotype as indicated by asterisks. *n* = 18 mice/48 responses for TASK^+/+^, *n* = 12 mice/31 responses for TASK^−/−^, *n* = 10 mice/30 responses for TASK^−/−^ with gavage of SPIR, *n* = 12 mice/36 responses for TASK^−/−^ with ICV injection of SPIR. **(D,E)** The mean change in SBP pressure (ΔSBP) and HR (ΔHR) in response to intravenous injection of SNP. *n* = 18 mice/41 responses for TASK^+/+^, *n* = 12 mice/34 responses for TASK^−/−^, *n* = 10 mice/30 responses for TASK^−/−^ with gavage of SPIR, *n* = 12 mice/33 responses for TASK^−/−^ with ICV injection of SPIR. ^*^p < 0.05, ^**^p < 0.01, ^***^p < 0.001 as indicated, one-way ANOVA with Tukey’s multiple comparisons test. PE, phenylephrine; SNP, sodium nitroprusside.

**Figure 7:**
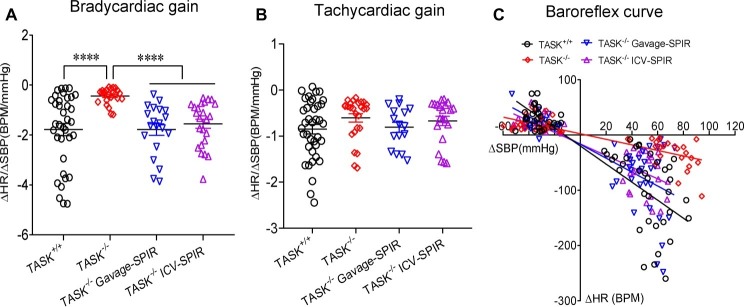
Impairment of baroreflex gain in TASK^−/−^ mice. The baroreflex gain was calculated based on the change in SBP (ΔSBP) and HR (ΔHR) during intravenous injection of PE (10 and 20 μg/kg) and during intravenous injection of SNP (30 and 60 μg/kg) in TASK^+/+^ mice (*n* = 18 mice/70 responses), TASK ^−/−^ mice (*n* = 12 mice/50 responses), TASK^−/−^ mice with gavage of SPIR (*n* = 10 mice/48 responses), and TASK^−/−^ mice with ICV injection of SPIR (*n* = 12 mice/45 responses), respectively. **(A,B)** Bradycardiac rather than tachycardiac gain was significantly decreased in TASK^−/−^ mice relative to TASK^+/+^ mice. ^****^p < 0.0001 as indicated by Kruskal-Wallis test followed by Dunn’s multiple comparisons test. **(C)** The baroreflex curve showing individual data points for the change in heart rate (ΔHR) in response to induced changes in SBP (ΔSBP).

Having found the presence of dysautonomia in TASK^−/−^ mice, we next assessed arterial baroreflex function in anesthetized mice. Figure 6A shows typical responses of BP and HR to PE; the pressor response to PE (10, 20 μg/kg) was remarkably enhanced in TASK^−/−^ mice compared to TASK^+/+^ mice and such effects were abolished by SPIR (Figure 6B). The PE-stimulated reflex control of HR decrease was significantly attenuated in TASK^−/−^ mice relative to TASK^+/+^ mice; administration of SPIR caused variable effects on bradycardiac responses (Figure 6C). However, in response to SNP, both genotypes exhibited similar decrease in SBP (Figure 6D) and variable tachycardiac responses (Figure 6E). Next, we normalized these data and calculated the baroreflex gain using ΔHR/ΔSBP. As shown in Figure 7, TASK^−/−^ mice displayed reduced bradycardiac gain of baroreflex compared to the counterparts (*p* < 0.0001, Figure 7A) and blockade of MRs disinhibited the depressed bradycardiac gain (*p* < 0.0001, Figure 7A). However, no obvious difference was observed in tachycardiac gain between two genotypes (Figure 7B). Therefore, TASK^−/−^ mice exhibited an asymmetrical impairment of the baroreflex curve and the damaged function of baroreflex could be to different degree rescued when systemically or centrally blocking MRs (Figure 7C).

## Discussion

Here, we present evidence of a significant contribution of MR activation to hyperaldosteronism-associated hypertension in a TASK^−/−^ mouse line that represents a model of PA. Either systemic or ICV blockade of MRs significantly decreases elevated arterial pressure in this PA model. This hypertensive mouse line exhibits amplified sympathetic and unchanged parasympathetic control of cardiovascular activity, and a diminished bradycardiac gain of the baroreflex, all indicative of an impaired baroreflex function.

### Hypertension in the TASK^−/−^ Mouse Line

The Aldo/MR signaling pathway has been implicated in autonomic regulation of cardiovascular activity (Gomez-Sanchez and Gomez-Sanchez, 2012). In those studies, either acute (minute to hour) or chronic (day to week) systemic or ICV infusion of Aldo increased BP and such effects were blocked in the presence of MR blockade. Note that these experimental models do not fully replicate the hallmark of PA because there might be cellular, molecular, and genomic differences in Aldo-targeting organ between short- and relatively long-lasting treatments with Aldo. The mechanism revealed using these animal models is unlikely fully responsible for the development of hypertension in animals and human with PA. Strikingly, the TASK^−/−^ mouse line serves as a suitable model to investigate hypertensive causes of PA (Davies et al., 2008). In this PA model, the sustained high level of Aldo is postulated to result in long-lasting damage to Aldo-targeting cells. The present data demonstrate a mild hypertension in TASK^−/−^ mice, manifesting elevated hourly BP over 24 h, 24-h average BP and day/night BP. Combined with the prior findings (Davies et al., 2008; Guagliardo et al., 2012, 2019), we confirm that the present data obtained in this PA model are solid and reliable to help understand causative factors of hypertension.

### Mineralocorticoid Receptor-Dependent Hypertension

TASK channels are present in cardiovascular reflex pathways, such as petrosal and nodose ganglion, nucleus tractus solitarius (NTS), and ventrolateral medulla (Talley et al., 2001). TASK channels play a critical role in the maintenance of membrane potential and excitability (Talley et al., 2003). We mainly ascribe the hypertensive mechanism to Aldo excess and activation of MR rather than TASK channel knockout in this PA model based on the following reasons. First, 24-h concentrations of plasma Aldo measured in the present study were always higher in TASK^−/−^ mice relative to control mice, consistent with 24-h dynamic change in BP in both genotypes, although the circadian rhythm of BP and plasma Aldo was not exactly parallel. Second, both systemic and central administration of the MR antagonist greatly reduced elevated BP to control level in TASK^−/−^ mice, suggesting that the contribution of Aldo/MR signaling played a critical role in the development of hypertension. Third, both systemic and central blockade of MRs to a large extent rescued the impaired baroreflex gain that is critical for regulation of rapid changes of BP levels. Finally, Aldo/MR signaling not only modulates salt and water homeostasis, but also plays an important role in the regulation of BP through modulating vessel responsiveness to various vasoactive factors. Therefore, high level of Aldo contributes to vascular dysfunction, all in turn promoting hypertension (Gomez-Sanchez et al., 2010). Although herein we did not test the effect of Aldo/MR signaling on vascular tone in this PA model, the antihypertensive effect of systemic blockade of MRs most likely comprises the peripheral component. Collectively, the onset of hypertension is closely associated with Aldo/MR signaling pathway in this PA animal model.

### Impairment of Baroreflex Contributes to Hypertension

Activation of the arterial baroreflex during rapid elevation of BP results in enhancement of parasympathetic but inhibition of sympathetic drives, with the result of reduction of HR and dilation of peripheral arteries, thereby buffering the rise in arterial pressure. The sustained impairment of baroreflex contributes to increased cardiovascular risk in hypertensive patients (Ormezzano et al., 2008). Hence, the arterial baroreflex is an important homeostatic mechanism. In the present study, we demonstrate evidence of enhanced sympathetic but not parasympathetic control of HR and BP in TASK^−/−^ mice, indicative of an autonomic output imbalance. We also provide evidence of an asymmetrical impairment of baroreflex in this PA model, manifesting that bradycardiac rather than tachycardiac gain of the baroreflex was significantly attenuated and the ability to buffer a pressor response was obviously compromised. Moreover, an impaired baroreflex function was observed not only in healthy humans following systemic infusion of Aldo (Schmidt et al., 2005; Monahan et al., 2007), but also in patients with PA (Veglio et al., 1999). Therefore, the hypertensive mechanism is closely correlated with dysfunctional baroreflex.

Our present findings indicate that chronic oral administration of SPIR, a competitive antagonist of MRs, not only decreased elevated BP, but also improved impaired gain of baroreflex in TASK^−/−^ mice, consistent with a previous report showing that oral administration of low-dose SPIR attenuated sympathetic drive and improved baroreflex function in rats with heart failure (Grassi et al., 1995). All these studies underscore the concept that systemic SPIR can effectively block both peripheral and central MRs. Moreover, this concept is also underpinned by the present evidence that chronic central application of SPIR greatly improved dysautonomia and baroreflex function in TASK^−/−^ mice. Based on the present effect of central MRs, the neurogenic component is partly responsible for MR-dependent dysautonomia and impaired baroreflex in TASK^−/−^ mice. This notion is also reinforced by prior reports demonstrating that the central Aldo/MR signaling plays an important role in regulation of sympathetic drive and baroreflex in the NTS, rostral ventrolateral medulla, and hypothalamic paraventricular nucleus (Masuda et al., 2010; Nakagaki et al., 2012; Zhang et al., 2012; Lincevicius et al., 2015). Due to lack of experimental data in the present study, the contribution of peripheral MRs is not excluded. Interestingly, MR specificity for aldosterone in mineralocorticoid target cells is thought to be determined extrinsically by the enzyme 11β-hydroxysteroid dehydrogenase type 2 (HSD2) (Gomez-Sanchez, 2011). In addition, MR and HSD2 have been shown to colocalized in the NTS (Geerling et al., 2006) and global deletion of HSD2 in mice resulted in impaired baroreflex (Evans et al., 2016). However, it remains to be determined whether HSD2 neurons in the NTS are required for MR-mediated impairment of baroreflex in TASK^−/−^ mice.

Note that an early study indicated that there was equally little change in MAP and HR in sino-aortic denervation dogs when compared to the control group. When baroreceptor and cardiopulmonary receptor afferents were instantaneously interrupted, both BP and HR rose to significantly higher levels (Persson et al., 1988). This does not necessarily mean that the impaired baroreflex contributes to hypertension.

## Summary

**Figure 8:**
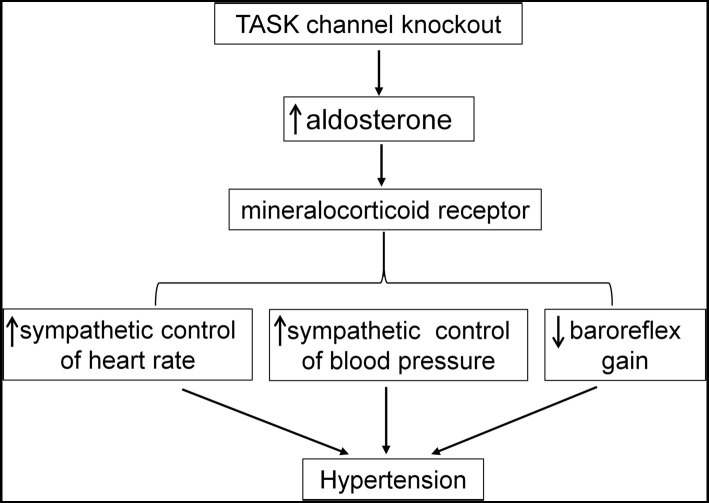
Summary of the mechanism underlying hyperaldosteronism-associated hypertension. Genetic deletion of TASK channels results in hyperaldosteronism. Then, overactivation of MR by Aldo contributes to enhanced sympathetic control of HR and BP and impairment of baroreflex, finally leading to elevated BP.

Here, we demonstrate hyperaldosteronism-related and MR-dependent hypertension in a TASK^−/−^ mouse line that represents a PA model. Enhanced sympathetic drive and impaired baroreflex are suggested to contribute to the development of hypertension. Figure 8 is a schematic summary of the mechanism underlying hyperaldosteronism-associated hypertension. In addition to systemic treatment, central blockade of MRs is also found to exert an antihypertensive effect probably through an attenuation of sympathetic drive and disinhibition of the baroreflex. Moreover, we validate the centrally antihypertensive effect of MR antagonists. These findings consolidate the understanding of the mechanism underlying hyperaldosteronism-associated hypertension.

## Data Availability Statement

All datasets generated for this study are included in the article/supplementary material.

## Ethics Statement

The animal study was reviewed and approved by Animal Care and Ethical Committee of Hebei Medical University. Written informed consent was obtained from the owners for the participation of their animals in this study.

## Author Contributions

LS, FY, XW, RW, KL, YT, and ZG acquired, analyzed, and interpreted the data. XZ coordinated the experiment. SW designed the studies and drafted the article. All authors revised the article critically for important intellectual content. All authors have approved the final version of the manuscript and agree to be accountable for all aspects of the work.

### Conflict of Interest

The authors declare that the research was conducted in the absence of any commercial or financial relationships that could be construed as a potential conflict of interest.
